# *Helicobacter pylori* infection and low dietary iron alter behavior, induce iron deficiency anemia, and modulate hippocampal gene expression in female C57BL/6 mice

**DOI:** 10.1371/journal.pone.0173108

**Published:** 2017-03-29

**Authors:** Monika Burns, Aldo Amaya, Caroline Bodi, Zhongming Ge, Vasudevan Bakthavatchalu, Kathleen Ennis, Timothy C. Wang, Michael Georgieff, James G. Fox

**Affiliations:** 1 Division of Comparative Medicine, Massachusetts Institute of Technology, Cambridge, Massachusetts, United States of America; 2 The Broad Institute of Harvard and MIT, Cambridge, Massachusetts, United States of America; 3 Division of Neonatology, Department of Pediatrics, University of Minnesota Medical School, Minneapolis, Minnesota, United States of America; 4 Department of Medicine, Columbia University, New York, New York, United States of America; 5 Department of Biological Engineering, Massachusetts Institute of Technology, Cambridge, Massachusetts, United States of America; Pennsylvania State University College of Medicine, UNITED STATES

## Abstract

*Helicobacter pylori (H*.*pylori)*, a bacterial pathogen, is a causative agent of gastritis and peptic ulcer disease and is a strong risk factor for development of gastric cancer. Environmental conditions, such as poor dietary iron resulting in iron deficiency anemia (IDA), enhance *H*.*pylori* virulence and increases risk for gastric cancer. IDA affects billions of people worldwide, and there is considerable overlap between regions of high IDA and high *H*.*pylori* prevalence. The primary aims of our study were to evaluate the effect of *H*.*pylori* infection on behavior, iron metabolism, red blood cell indices, and behavioral outcomes following comorbid *H*. *pylori* infection and dietary iron deficiency in a mouse model. C57BL/6 female mice (n = 40) were used; half were placed on a moderately iron deficient (ID) diet immediately post-weaning, and the other half were maintained on an iron replete (IR) diet. Half were dosed with *H*.*pylori* SS1 at 5 weeks of age, and the remaining mice were sham-dosed. There were 4 study groups: a control group (-*Hp*, IR diet) as well as 3 experimental groups (-*Hp*, ID diet; +*Hp*, IR diet; +*Hp*,ID diet). All mice were tested in an open field apparatus at 8 weeks postinfection. Independent of dietary iron status, *H*.*pylori* -infected mice performed fewer exploratory behaviors in the open field chamber than uninfected mice (p<0.001). Hippocampal gene expression of myelination markers and dopamine receptor 1 was significantly downregulated in mice on an ID diet (both p<0.05), independent of infection status. At 12 months postinfection, hematocrit (Hct) and hemoglobin (Hgb) concentration were significantly lower in +*Hp*, ID diet mice compared to all other study groups. *H*.*pylori* infection caused IDA in mice maintained on a marginal iron diet. The mouse model developed in this study is a useful model to study the neurologic, behavioral, and hematologic impact of the common human co-morbidity of *H*. *pylori* infection and IDA.

## Introduction

The Gram-negative pathogen, *Helicobacter pylori*, infects over 50% of the world’s population, and was classified as a group I carcinogen in 1994 by the International Agency for Research on Cancer [1]. *H*. *pylori* infection causes acute and chronic inflammatory responses in the stomach, which can result in a multitude of disorders including: chronic gastritis, atrophic gastritis, peptic ulcers, gastric cancer, and iron deficiency anemia (IDA) [2–7]. The prevalence of *H*. *pylori* infection in developed countries has decreased with improved sanitary conditions and continued therapeutic intervention [5]. In spite of this progress, *H*. *pylori* prevalence rates remain as high as >90% in some developing countries, which corresponds to higher risk of gastric cancer in some subpopulations [8]. *H*. *pylori* also has been postulated to play a role in chronic neurologic disorders. A prevalent theory as to how *H*. *pylori* may contribute to neurologic disease is that *H*. *pylori*-induced cytokines and chemokines cause systemic and central nervous system (CNS) inflammation and dysfunction[9].

*H*. *pylori* infection and iron deficiency anemia (IDA) are both prevalent in the developing world, and overlapping diseases represent an increased risk for co-morbidity of these two conditions [8,10,11]. Iron is an essential micronutrient with many critical functions in the body, including as an important component of heme compounds that are crucial in the process of oxygen delivery to cells [12]. Iron deficiency (ID) is the most prevalent micronutrient deficiency in many demographic groups worldwide, including children and women of childbearing age. Consumption of a marginal iron diet is one of the most common causes of dietary ID in developing countries [10,13,14] When ID is present during important developmental periods, such as fetal and early postnatal stages, it causes cognitive and socio-emotional deficits in infants and children that persist into adulthood [15–17]. Treatment of IDA during childhood with iron supplementation often fails to prevent the occurrence of deficits, including poor performance IQ scores, visual motor integration problems and gross and fine motor performance deficits, at later developmental stages [18]. The negative neurodevelopmental effects of early-life ID seen in human patients have been substantiated in rodent models [19–21].

Recent basic and clinical research has focused on elucidating the relationship between *H*. *pylori* infection and IDA, and how one condition may impact the other. Four recent meta-analyses have concluded that *H*. *pylori* infection is a causative factor in the development of IDA in human patients, with proposed mechanisms including: decrease in absorption of dietary iron due to hypochlorhydria caused by *H*. *pylori* infection, gastrointestinal blood loss, and enhanced uptake and sequestration of iron by *H*. *pylori* [4,22–24]. A previous study conducted in our lab revealed that male INS-GAS/FVB mice infected with *H*. *pylori* develop anemia and have significantly lower serum ferritin concentrations than uninfected control mice at 7–8 months postinfection. Interestingly, this occurred in spite of continuous intake of an iron-replete rodent diet. Molecular analysis of whole brain tissue revealed that chronic *H*. *pylori* infection of INS-GAS mice resulted in reduced expression of genes involved in dopamine metabolism, myelination, and maintenance of synaptic plasticity; all changes characteristic of brain iron deficiency [25]. These findings prompted the current study in C57BL/6 mice, a strain commonly used as a background strain for behavioral studies. This study is the first to evaluate the effects of *H*. *pylori* infection on hippocampal gene expression in mice. The goals of this study were to determine: 1) the effect of *H*. *pylori* infection on mouse behavior, 2) the effect of chronic *H*. *pylori* infection on expression of genes related to myelination in the hippocampus, 3) how chronic *H*. *pylori* infection and concurrent marginal dietary iron deficiency affect dopamine metabolism in the hippocampus, and 4) the impact of chronic *H*. *pylori* infection and marginal dietary iron on systemic iron homeostasis and red blood cell indices.

## Materials and methods

### Animals

The Massachusetts Institute of Technology (MIT) Committee on Animal Care approved the use of mice in this study (Animal Welfare Assurance #A3125-01). All mice were euthanized via carbon dioxide inhalation, in accordance with the American Veterinary Medical Association Euthanasia Guidelines [26]. Female C57BL/6NCrl mice obtained from Charles River Laboratories (Wilmington, MA) were used and maintained on purified diets (either iron-replete or moderately iron-deplete) from immediately post-weaning throughout the duration of the study. Female mice were chosen as a model for this study due to the fact that they develop a more severe proinflammatory Th1-biased response than male mice when infected with *H*. *pylori*, and C57BL/6 mice of both sexes provide reliable models for behavioral studies [27,28]. There were a total of 4 treatment groups (1) -*Hp*, IR; 2)+*Hp*, IR; 3)-*Hp*, ID; 4) +*Hp*, ID), with 7–11 mice per treatment group at study initiation. All mice were maintained in an AAALAC International-accredited facility and were seronegative for: mouse hepatitis virus, Sendai virus, rotavirus (EDIM), pneumonia virus of mice, Theiler’s encephalomyelitis virus, reovirus, lymphocytic choriomeningitis virus, ectromelia virus, polyomavirus, K virus, carbacillus, mouse cytomegalovirus, mouse parvovirus, and minute virus of mice. Mice were also negative for both endo- and ectoparasites, including *Syphacia & Aspicularis* spp, as well as *Salmonella* spp, *Citrobacter rodentium*, & *Helicobacter* spp. Mice were socially-housed, with two to five mice housed in each standard mouse cage. Mice were housed on heat-treated hardwood bedding (P.J. Murphy Forest Products Corporation; Montville, NJ) with one sterile cotton fiber Nestlet (Ancare) per cage in polycarbonate caging with filter tops, were maintained on a 12:12 light/dark light cycle, and were provided customized purified diet and reverse osmosis chlorinated water ad libitum.

### Diet

Mice were allowed ad libitum access to one of two customized purified diets created by Research Diets Inc (New Brunswick, NJ) at approximately 23 days of age (immediately post-weaning). This rodent life stage was selected to model a period of intensive growth in the human brain [29].Dietary iron content was independently verified via ICP Emission Spectrometry performed by Covance Laboratories (Princeton, NJ). The iron concentration of the iron-replete (IR) diet ranged from 47.8–48.7 ppm, and the iron concentration of the moderately iron-deplete (ID) diet ranged from 7.57–8.69 ppm.

### Experimental infection

At 5–5.5 weeks of age, female mice were orally gavaged with 1 X 10^8^ colony forming units of *H*. *pylori* SS1 in 200 μl of sterile freeze media every other day for a total of 3 doses, or were sham-dosed with sterile freeze media following the same timeline. Dosing at this age was selected for infection since infecting neonatal mice with *H*. *pylori* results in the development of tolerance to the pathogen, which precludes development of chronic inflammation [30].

### Open field task

Behavioral testing was conducted at 8 weeks postinfection (13 weeks of age). Locomotor activity and behavioral responses to an unfamiliar environment were measured using the open field test. Mice were individually placed into a Plexiglas testing chamber measuring 40 cm x 40 cm x 30 cm. Mice were placed in the center of the testing chamber initially, and allowed to explore the area for 60 minutes. Motor activity was detected and tracked by infrared photobeam sensors. Individual mouse activity was analyzed by a VersaMax animal activity monitoring system (AccuScan Instruments; Columbus, OH). Parameters measured included: total distance travelled, ambulatory activity, ambulatory episodes, horizontal activity, vertical activity, and total time spent in each area. At the end of the task, all mice were returned to their home cage. Equipment was thoroughly cleaned with Quatricide disinfectant. All behavioral testing took place between 9am and 2pm. In the behavioral testing room, soft lighting provided by a floor lamp containing a single 40W fluorescent light bulb was present during task preparation and chamber cleaning.

### Complete blood count

Serial blood samples (< 200μl) were collected at 2, 3, 6, &10 months of age via submandibular venipuncture, and immediately placed into 1 ml EDTA-coated anticoagulant blood collection tubes. Samples were processed by a HemaVet 950 veterinary hematology analyzer (Drew Scientific, Oxford, CT) at the MIT DCM diagnostic laboratory. Whole blood samples (1 mL) were collected at necropsy from anesthetized mice via cardiac puncture, and immediately placed into 1 mL EDTA-coated anticoagulant blood collection tubes. Samples were processed within 4 hours of collection at the Massachusetts General Hospital Center for Comparative Medicine Clinical Pathology lab (Boston, MA). Complete Blood Count (CBC) analysis was performed on a HESKA HemaTrue Veterinary Analyzer (Loveland, CO), using an impedance-based method for counting white blood cells, red blood cells, and platelets. Hemoglobin was measured on the HemaTrue Analyzer using a cyanide-free method and measured by a spectrophotometer set to 535 nm.

### Serum ferritin

Serum ferritin concentrations were measured using a mouse specific enzyme-linked immunosorbent assay (ELISA) kit (Kamiya Biomedical, Seattle, WA) according to the instructions provided by the manufacturer. A positive internal control, serum containing a known quantity of ferritin, was included in the commercial kit, and analyzed concurrently with experimental samples. The R2 value of the standard curve was 0.998. All samples were analyzed in duplicate.

### Necropsy

Necropsies were performed on all mice at 12 months postinfection. Whole brains were collected immediately post-mortem, and the hippocampus was dissected bilaterally and immediately submerged in liquid nitrogen. All samples were ultimately transferred to and stored at -80°C. Stomach and proximal duodenum were sectioned beginning at the greater curvature, and gastric linear sections were collected for histopathology. Other stomach sections were used for DNA and RNA extraction to evaluate mRNA expression and *H*. *pylori* colonization level, as previously described [25]. Stomach tissue samples (linear strips from the squamo-columnar junction through the proximal duodenum) and liver tissue samples collected for DNA and RNA extraction were flash-frozen in liquid nitrogen and stored at -80°C. Sterile razor blades were used for stomach and duodenal sectioning. Sections of stomach and liver were collected, trimmed, and fixed overnight in 10% neutral buffered formalin. All tissues were processed, embedded in paraffin, cut into 4-μ m-thick sections, and stained with hematoxylin and eosin.

### Histopathologic evaluation and lesion scoring

All tissues collected at necropsy were evaluated by a board-certified comparative pathologist (V.B.) who was blinded to both treatment groups and sample identity. Gastric lesions were scored on an ascending scale from 0 to 4 using previously established criteria. Lesions related to inflammation, epithelial defects, oxyntic atrophy, epithelial hyperplasia, pseudo-pyloric metaplasia, mucus metaplasia, and dysplasia, were scored. Scores for each individual category were added to generate an overall gastric histopathologic assessment index (GHAI) [31,32].

### Real time quantitative PCR

mRNA expression for target genes was assessed in both gastric and liver tissues. Stomachs were incised along the greater curvature, opened, and sectioned into three linear strips. Total RNA from liver and stomach was prepared using Trizol reagent (Life Technologies), according to the manufacturer’s instructions. Two μg of total RNA from each sample was converted to cDNA using a High-Capacity cDNA Reverse Transcriptase Kit (Applied Biosciences). Relative expression of hepcidin antimicrobial peptide (*Hamp*), bone morphogenic peptide 4(*Bmp4*), divalent metal transporter (*Slc11a2*; hereafter referred to as *Dmt1*), as well as levels of inflammatory cytokines including: tumor necrosis factor alpha (*TNFα*), interleukin 1 beta (IL1β), interleukin 17 alpha (IL17α), and interferon gamma (IFNγ) mRNA were measured by qPCR using commercially available primer/probes (TaqMan Gene Expression Assays) in the 7500 Fast Sequence Detection System. CT values were normalized to an endogenous control, glyceraldehyde-3-phosphate dehydrogenase mRNA, and were expressed as relative fold-change compared to sham-dosed animals using the comparative CT method. Expression levels of target genes were also analyzed in hippocampal tissue. Total RNA from hippocampal tissue was extracted (RNAqueous kit, Lifetechnologies; Carlsbad, CA) and first strand cDNA was generated using 2 μg of RNA (high capacity RNA-to cDNA kit, Lifetechnologies; Carlsbad, CA). Relative mRNA expression of myelin basic protein (*Mbp*), proteolipid protein 1 (*Plp1)* and dopamine receptor 1 (*D1r*) was evaluated using qPCR [33,34]. Commercially available primer/probes (Lifetechnologies; Carlsbad, CA) and Taqman master-mix (Roche Life Science; Indianapolis, IN) were used on a StratageneMX300P qPCR system (Agilent Technologies; Santa Clara, CA). Samples were assayed in duplicate and normalized against ribosomal protein S18.

### Colonization levels of *H*. *pylori* SS1

DNA was extracted from stomach tissue using a High Pure PCR Template Preparation Kit (Roche Diagnostics, Indianapolis, IN). Colonization levels of *H*. *pylori* SS1 within the gastric mucosa were quantified using *H*. *pylori* DNA-specific primer/probes in the 7500 Fast Sequence Detection System (Applied Biosystems) as previously described [35]. To quantify murine DNA in the respective samples, each was probed with commercial 18S rRNA gene-based primer/probes (Life Technologies) as previously published [35]. Copy numbers of *H*. *pylori* were measured using per μg of mouse DNA.

### Statistical analysis

All statistical analyses were performed using GraphPad Prism Version 7 (GraphPad Software Inc.). Distribution and variance were evaluated for all samples, and statistical analyses were performed using parametric methods after assumptions of normality and equivalence of variance were met. One-way analysis of variance (ANOVA) or Student’s t-tests were used, and multiple comparisons tests (Tukey’s) were performed following ANOVA. Nonparametric tests were used for comparison of mean gastric histopathology scores across groups, and Dunn’s multiple comparisons tests were used for post-hoc analysis. Statistical significance was designated as a p-value < 0.05.

## Results

### Female mice perform fewer exploratory behaviors in the open field chamber after acute *H*. *pylori* infection

**Fig 1:**
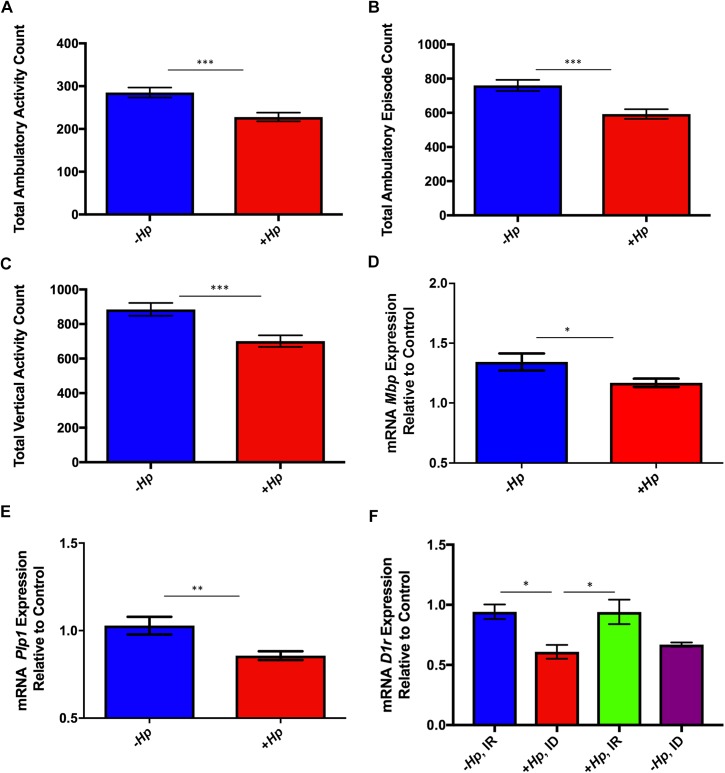
**(A-F). *H*. *pylori-*infected mice performed fewer exploratory behaviors in the open field chamber and hippocampal expression of genes related to myelination and dopamine metabolism was altered by *H*. *pylori* infection and dietary iron content.** (A-C) *H*. *pylori*-infected mice had significantly lower total ambulatory activity, total ambulatory count, and total vertical activity count compared to uninfected mice independent of dietary iron status at 8 weeks postinfection. (D) Expression of hippocampal genes related to myelination was downregulated in *H*. *pylori* infected female mice at 12 months postinfection. Expression of both *Mbp* (D) and *Plp1* (E) mRNA was significantly downregulated in *H*. *pylori*-infected mice compared with uninfected controls. (F) At 12 months postinfection, expression of *D1r* was significantly downregulated in *H*. *pylori* -infected mice on ID diet compared to all mice on IR diet. All data shown as mean + SEM. (*) = p<0.05, (**) = p<0.01, (***) = p<0.001.

Ambulatory activity and episodes, as well as vertical activity, are considered exploratory behaviors of mice in an open field chamber. Counts of these exploratory behaviors were significantly lower in *H*. *pylori-*infected C57BL/6 female mice compared to uninfected mice at 8 weeks postinfection (all p<0.001; Fig 1A–1C). At 8 weeks post-*H*. *pylori* infection, no effect of dietary iron status was noted, as evidenced by normal hemogram results at the 8 week postinfection time point.

### Chronic *H*. *pylori* infection and marginal iron diet altered hippocampal gene expression in female mice

Hippocampal gene expression of both myelin basic protein (*Mbp*) and proteolipid protein 1 (*Plp1*) was significantly downregulated in *H*. *pylori*-infected female mice compared to sham-dosed controls at 12 months postinfection (0.01< p <0.05; Fig 1D & 1E). Both *Mbp* and *Plp1* play a critical role in hippocampal axonal myelination. Dietary iron did not affect expression of *Mbp* or *Plp1*. A significant effect of treatment group was found on hippocampal expression of dopamine receptor 1 (*D1r*), an important component of the CNS dopamine system (p<0.01; Fig 1F). Multiple comparisons analysis revealed that *D1r* expression was significantly downregulated in adult *H*. *pylori*-infected mice on an ID diet compared to both infected and uninfected mice on an IR diet (both p<0.05; Fig 1F).

### Concurrent *H*. *pylori* infection dietary iron deficiency altered expression of hepatic iron regulatory genes

**Fig 2:**
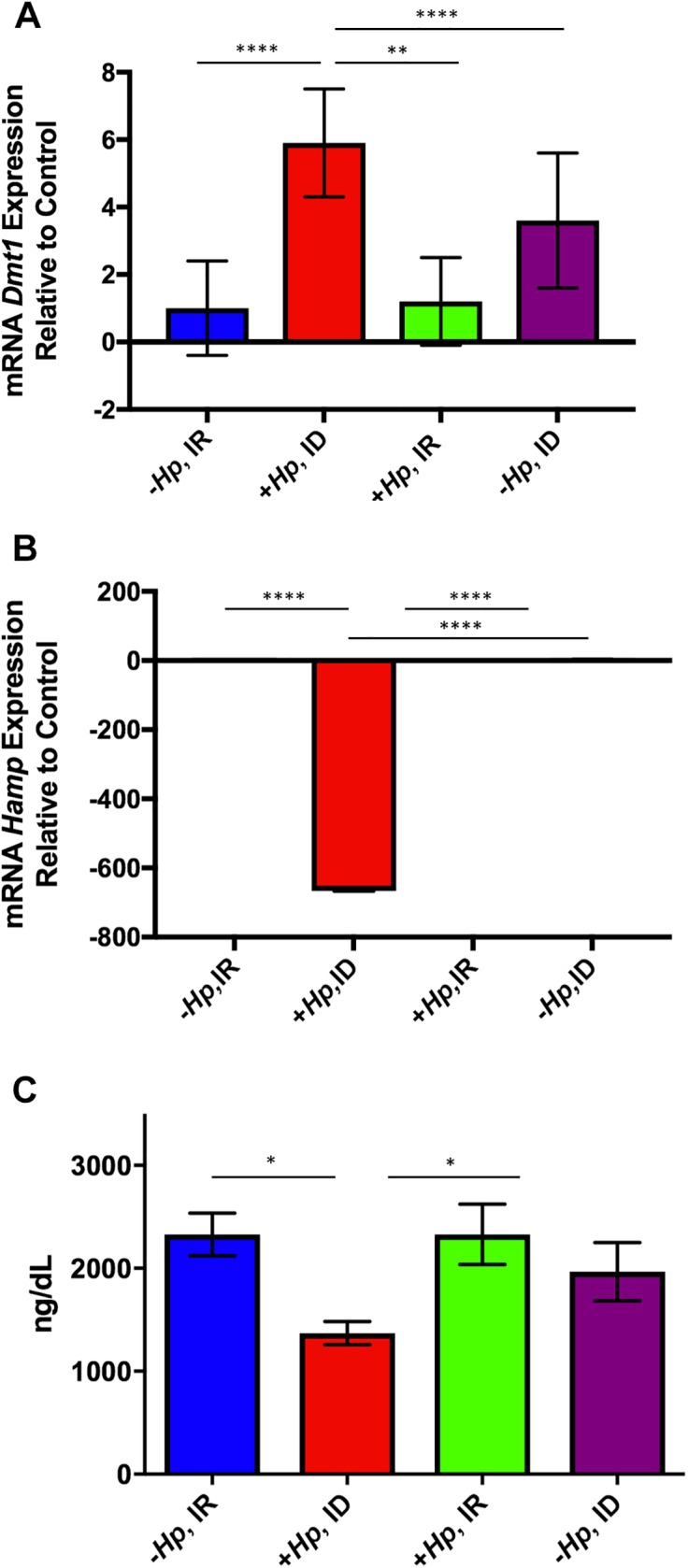
**(A-C). Hepatic *Dmt1* expression was upregulated while *Hamp* expression and serum ferritin were downregulated in *H*. *pylori*-infected mice on ID diet.** Hepatic *Dmt1* expression was upregulated and *Hamp* expression was downregulated in *H*. *pylori-*infected mice on ID diet. (A) *H*. *pylori-*infected mice had significantly increased expression of *Dmt1* compared to all other treatment groups. (B) Expression of *Hamp* in hepatic tissue was significantly downregulated in *H*. *pylori* -infected mice on ID diet compared to all other study groups. (C) Concurrent *H*. *pylori* infection and dietary ID decreased serum ferritin levels in female mice. *H*. *pylori*-infected mice on ID diet had significantly lower serum ferritin levels than both uninfected mice on IR diet and *H*. *pylori*-infected mice on IR diet. All data shown as mean + SEM. (*) = p<0.05, (**) = p<0.01, (****) = p<0.000.

Expression of divalent metal transporter 1 (*Dmt1*), an important iron transporter of many types of cells, was significantly upregulated in *H*. *pylori-*infected mice on ID diet compared to all other treatment groups (0.001< p<0.01); Fig 2A). Expression of hepcidin (*Hamp*) was significantly downregulated in *H*. *pylori-*infected mice on ID diet compared to all other treatment groups (all p<0.001; Fig 2B). The liver is the primary source of hepcidin antimicrobial peptide, the body’s master iron regulatory protein. No effect of treatment group was noted on hepatic gene expression of bone morphogenic protein 4 (*Bmp4)* (data not shown).

### Concurrent *H*. *pylori* infection and dietary iron deficiency depleted serum ferritin stores at 12 months postinfection

At necropsy, mice chronically infected with *H*. *pylori* on ID diet had significantly lower serum ferritin than uninfected and *H*. *pylori*-infected mice on IR diet (both p<0.05; Fig 2C). Ferritin is critical iron-binding protein, and iron bound to ferritin makes up a large proportion of the total iron storage pool of the body.

### Gastric expression of inflammatory cytokines was upregulated in *H*. *pylori*-infected female mice

**Fig 3:**
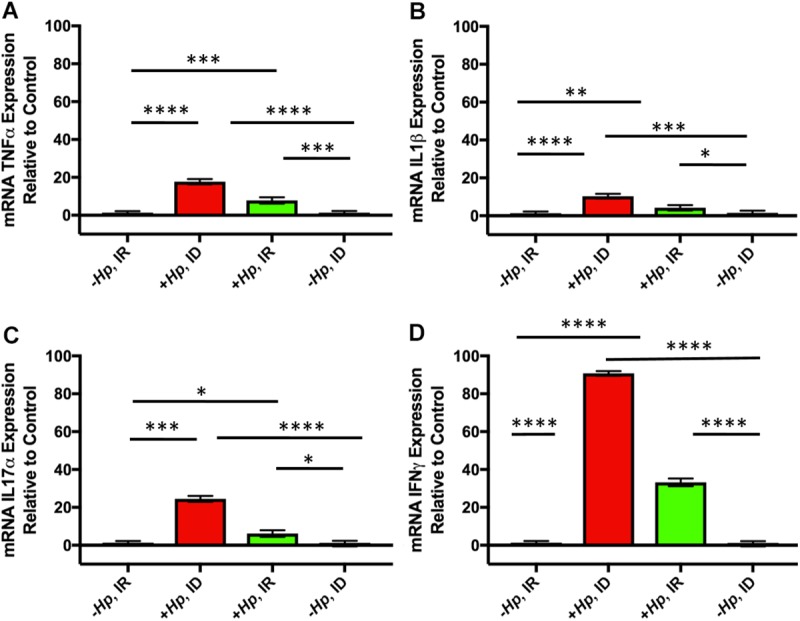
Chronic *H. pylori* infection upregulated gastric expression of inflammatory cytokines in female mice. Gastric expression of tumor necrosis factor alpha (TNFα) (A), interleukin one beta (IL1β) (B), interleukin 17 alpha (IL7α) (C), and interferon gamma (IFNγ) (D) was significantly upregulated in *H*. *pylori* infected mice compared to uninfected mice. All data shown as mean + SEM. (*) = p<0.05, (**) = p<0.01, (***) = p<0.001, (****) = p<0.0001.

Gastric expression of tumor necrosis factor alpha (TNFα), interleukin one beta (IL1β), interleukin 17 alpha (IL7α), and interferon gamma (IFNγ), was significantly upregulated in *H*. *pylori* infected mice compared to both groups of uninfected mice (0.05<p<0.0001; Fig 3A–3D). However, no significant differences in gastric inflammatory cytokine expression were noted between *+Hp*, ID and +*Hp*, IR treatment groups (Fig 3A–3D).

### *H*. *pylori-*infected female mice on marginal iron diet had lower hemoglobin concentration than control mice by 3 months postinfection

**Fig 4:**
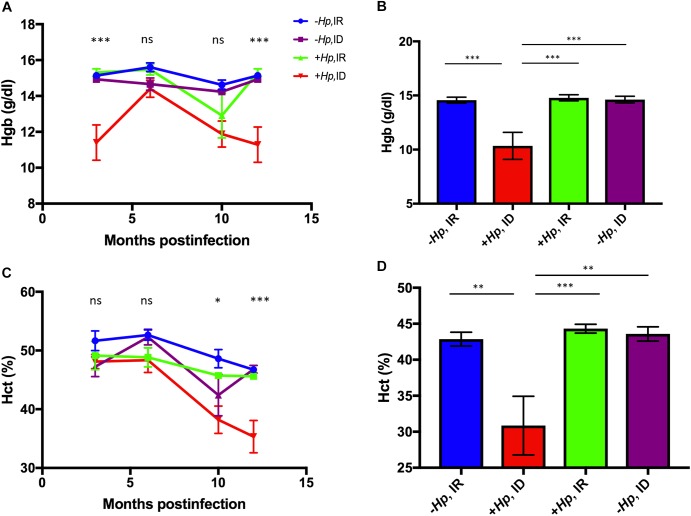
**(A-D). Hematocrit and hemoglobin concentration were lower in *H*. *pylori-*infected mice on ID diet.** (A) Mean hemoglobin (Hgb) differed among treatment groups at multiple sampling time points. A significant treatment effect on Hgb concentration was noted at the first sampling time point (3 months postinfection). At necropsy (B), *H*. *pylori -*infected mice on ID diet had lower Hgb than all other treatment groups. (C) Mean Hct was significantly different among treatment groups at both 10 and 12 months postinfection. (D) *H*. *pylori-*infected mice on ID diet had significantly lower mean Hct than all other groups at the time of necropsy. All data shown as mean + SEM. (*) = p<0.05, (**) = p<0.01, (***) = p<0.001.

Low hemoglobin (Hgb) concentration and hematocrit (Hct) are commonly used in the clinical diagnosis of anemia. Significant differences in mean Hgb concentration among treatment groups were noted at early and late sampling time points (Fig 4A–4D). A significant treatment effect on mean Hgb concentration was noted at 3 months postinfection (p<0.001; Fig 4A). Post hoc multiple comparisons analysis revealed that the +*Hp*, ID treatment group had significantly lower mean Hgb concentration than the–*Hp*, IR (p<0.001), -*Hp*, ID (p<0.001), and +*Hp*, IR (p<0.0001) treatment groups at 3 months postinfection (data not shown). No significant treatment effect was noted among groups at 6 or 10 months postinfection. At 12 months postinfection, a significant treatment effect was identified (p<0.0001; Fig 4A); multiple comparison analysis revealed that mean Hgb concentration of *H*. *pylori-*infected female mice on ID diet was significantly lower than all other treatment groups (p<0.001; Fig 4B).

### Concurrent *H*. *pylori* infection and dietary iron deficiency lowered hematocrit in female mice by 10 months postinfection

No significant differences in mean Hct were noted until 10 months postinfection, at which point mean Hct was significantly lower in the +*Hp*, ID group than the–*Hp*, IR group (p<0.05; Fig 4C). At 12 months postinfection, a significant treatment effect was noted among groups (p<0.001; Fig 4C). Mean Hct of the +*Hp*, ID group was significantly lower than all other treatment groups (0.01<p<0.001; Fig 4D).

Both mean Hgb and mean Hct of mice in the *H*. *pylori-*infected mice on ID diet were significantly lower than all other treatment groups after 12 months of infection.

### Concurrent *H*. *pylori-*infection and moderate dietary ID decreased mean cellular volume of erythrocytes and increased red blood cell distribution width in mice at 12 months postinfection

**Fig 5:**
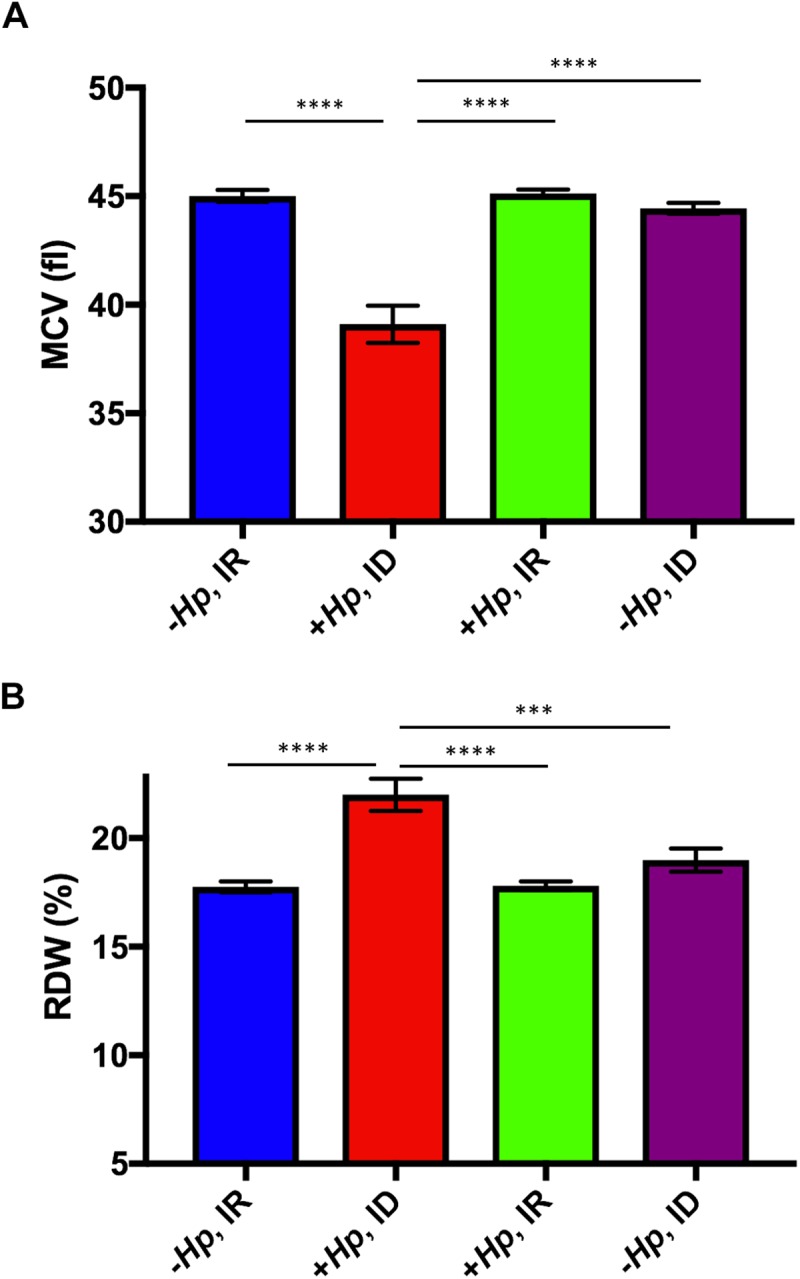
**(A&B). Concurrent *H*. *pylori infection and ID diet resulted* in lower mean cellular volume (MCV) and higher mean red cell distribution width (RDW) at 12 months postinfection** (A) Mean MCV of *H*. *pylori-*infected mice on ID diet was significantly lower than mean MCV of all other treatment groups. (B) Mean RDW of *H*. *pylori-*infected mice on ID diet was significantly greater than mean RDW of all other treatment groups. All data shown as mean + SEM (***) = p<0.001, (****) = p<0.0001.

Mean cellular volume (MCV) is a measurement of RBC size; red cells with reduced MCV are commonly noted on peripheral blood smears and/or complete blood counts as a result of iron deficient erythropoiesis. At 12 months postinfection with *H*. *pylori*, mice on an ID diet had significantly lower MCV than all other treatment groups (all p<0.0001; Fig 5A).

Red cell distribution width (RDW) is a parameter that measures variation in red blood cell size [36]. High RDW reflects considerable variation in red blood cell size, and is commonly documented in iron deficient patients. Mice infected with *H*. *pylori* for 12 months and maintained on ID diet had significantly higher RDW compared to all other treatment groups (0.0001< p< 0.001; Fig 5B).

### Histopathology

**Fig 6:**
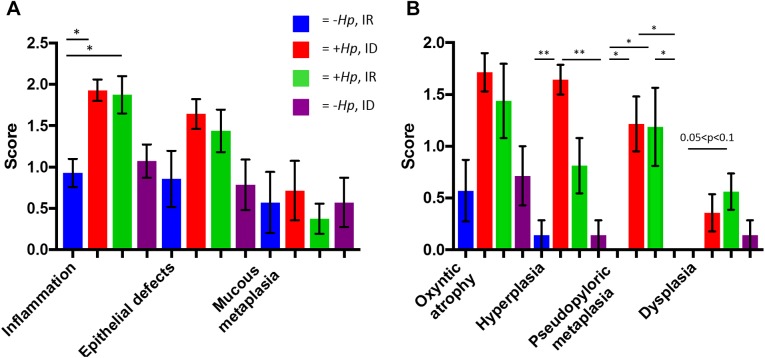
**(A&B). *H*. *pylori* infection increased gastric corpus histopathology scores.** *H*. *pylori-*infected mice had higher histopathology scores for(A) inflammation, (B) hyperplasia, pseudopyloric metaplasia. All data shown as mean + SEM. (*) = p<0.05, (**) = p<0.01, (****) = p<0.0001.

*H*. *pylori* SS1-infected mice had significantly higher inflammation, hyperplasia, and pseudopyloric metaplasia scores than uninfected mice (Fig 6A & 6B).

### *H*. *pylori* SS1 established persistent colonization in gastric tissue at 12 months postinfection

Colonization levels of gastric tissue by *H*. *pylori* SS1 were evaluated at necropsy (12 months postinfection). There were two mice in the +*Hp*, IR treatment group, and one mouse in the +*Hp*, ID treatment group that had lost colonization at the time of necropsy, and thus were excluded from statistical analysis. Mean relative colonization level of mouse gastric tissue in persistently *Hp-*infected mice on ID diet was 15503+6834 copies of *H*. *pylori/*μg of mouse DNA. Mean relative colonization level of mouse gastric tissue in persistently *Hp-*infected mice on IR diet was 1234+512.9 copies of *H*. *pylori/*μg of mouse DNA (0.05<p<0.1).

## Discussion

In this study, behavioral testing was conducted at 8 weeks postinfection, which corresponds to the active phase of inflammation caused by gastric *Helicobacter* spp. Infections in C57BL/6 mice [7,37] Female C57BL/6 mice infected with *H*. *pylori* performed fewer exploratory behaviors in the open field chamber than did sham-dosed mice. This lack of exploratory behaviors is consistent with increased anxiety [38,39]. No hematologic evidence of ID due to consumption of a marginal iron diet was documented at this early timepoint; thus we attributed these behavioral changes to active *H*. *pylori*-associated inflammation. While the open field task is used as a measurement of locomotion and anxiety, it is possible that discomfort due to gastric inflammation caused by *H*. *pylori* infection influenced behavioral task performance in this study. Multiple hypotheses have been proposed regarding how *H*. *pylori* infection affects the brain, including that of a causative relationship between *H*. *pylori*-associated inflammation and altered neuronal homeostasis [40]. The behavioral effects of murine GI infections, causing acute inflammation, have been studied previously as models of human disease. Murine *Citrobacter rodentium* infection used to model inflammatory bowel disease (IBD) produced anxiety-like behaviors in mice [41]. This study found that mice infected with *C*. *rodentium* demonstrated more anxiety-like behaviors compared to uninfected controls, as *C*. *rodentium-*infected mice spent significantly less time in the center zone of the open field chamber than control mice [41]. Thus, acute *H*. *pylori* infection, similar to infections of the lower bowel that cause inflammation, modulated behavioral task performance in mice, and can provide a robust animal model for the study of the effect of GI inflammatory disease on the brain and behavior in humans.

In addition to acute behavioral alterations, hippocampal expression of two genes related to myelination, *Mbp* and *Plp1*, was lower in mice chronically infected with *H*. *pylori* regardless of dietary iron status when compared to sham-dosed control mice. This indicates that *H*. *pylori* infection per se was an independent factor in altering hippocampal gene expression. Mbp and Plp1 are essential to formation of the myelin sheath in the mammalian central nervous system. Previous studies evaluating gene expression in the hippocampus have found that protein levels trend in the same direction as the corresponding gene transcripts [42]. In our previous study, expression of *Mbp* was significantly lower in brain tissue of *H*. *pylori-*infected INS-GAS mice compared to uninfected controls [25]. Protein analysis revealed a parallel decrease in Mbp levels, which were 28% lower in *H*. *pylori-*infected mice compared to uninfected control mice. It is possible that dietary iron status in this study did not affect expression of myelination genes due to the use of a post-weaning marginal dietary ID model instead of a gestational dietary ID model.

Hippocampal expression of *D1r* was lower in mice on marginal ID diet than in mice on IR diet, independent of infection status. The impact of ID/IDA on the dopamine system has been studied extensively, and it is well-documented in rat studies that dopamine receptor expression and protein density decrease under ID conditions [39,43] Brain region-specific gene analysis was chosen for this study because the hippocampus plays a crucial role in learning and the formation of spatial and recognition memory. Adequate early life iron is essential for normal neuronal development, sensorimotor function, and dopamine system function in humans and rodents [42,44,45]. The results of the current study were consistent with previously reported findings where mice on ID diet had decreased expression of *D1r* compared to mice on IR diet. This disruption of dopamine system homeostasis demonstrates that environmental factors that originate early in life, such as marginal dietary ID, may impact nervous system gene expression even in aged animals. Previous studies have shown that long-term dopamine system changes may be induced by dietary ID without brain tissue ID [39]. In this study, no difference in expression of *Dmt1*, an iron regulator, was noted among treatment groups at 12 months postinfection. This is likely due to lower iron demand in the adult brain compared to the neonatal and juvenile brain.

Mean Hct and Hgb were significantly lower in *H*. *pylori-*infected mice on ID diet compared to all other groups in this study, and were also lower than previously established normal hematologic values of C57BL/6 female mice [46]. The combination of low Hct, Hg, and MCV, and concurrent high RDW, is indicative of a microcytic, hypochromic iron deficiency anemia [36]. It is likely that both *H*. *pylori* infection and low systemic iron availability contributed to this outcome. *H*. *pylori* disrupts host cell polarity and derails host intracellular iron-trafficking *in vitro* [47]. *H*. *pylori* infection causes inflammation, which in turn upregulates expression of hepcidin, an antimicrobial peptide produced by the liver that regulates systemic iron metabolism [48]. Atrophic gastritis caused by *H*. *pylori* infection results in a loss of parietal cells, thereby decreasing acid secretion into the gastric lumen, and increasing gastric pH [6,49]. An elevation in pH limits the amount of dietary ferric iron that is reduced to the ferrous form of iron absorbable by enterocytes, which results in impaired iron absorption.

A post-weaning marginal iron diet of 7-8ppm was selected for this study because gestational ID has been associated with high morbidity in mice [50]. The marginal iron diet provided mice with sufficient iron for homeostatic functions, evidenced by the lack of systemic effects of ID for several months after study initiation. Interestingly, mean Hgb concentration was significantly lower in *+Hp*, ID mice than all other treatment groups at 3 months postinfection, and at 12 months postinfection, but not at 6 and 10 month timepoints. In a previous study that evaluated the effect of low dietary iron (8ppm Fe) in both male and female C57BL/6 mice after 30 weeks of *H*. *pylori* infection, mice on the low iron diet had lower serum iron, transferrin saturation, and hemoglobin than uninfected controls on low iron diet [51]. The results of the current study corroborated many of these findings, and extended analysis to a chronic timepoint of 12 months postinfection. Another study of female C57BL/6 mice infected with either *H*. *felis* and *H*. *pylori* demonstrated that only *H*. *felis*, which induces a more robust inflammation than *H*. *pylori* in mice, caused an acute drop in serum iron after 4 weeks of infection; however, red blood cell indices and chronic time points were not evaluated [52]. In hypergastrinemic male INS-GAS mice prone to accelerated development of gastric adenocarcinoma, infection with gastric *Helicobacter spp*. Causes anemia and depleted serum iron stores, as well as altered expression of gastric and liver genes involved in systemic iron homeostasis [25,53]. In the current study, serum ferritin was lower in the *H*. *pylori*-infected group on ID diet than both IR treatment groups at 12 months postinfection, demonstrating that chronic infection with *H*. *pylori* infection, coupled with dietary ID, impacted serum iron storage as well as red blood cell parameters.

*H*. *pylori* colonization persisted for 12 months in mice that were infected at 4–5 weeks of age, and the histopathologic changes caused by a 12-month *H*. *pylori* SS1 infection in mice documented in the current study were consistent with previous studies in C57BL/6 mice infected with *H*. *pylori* SS1 for 12 months [32]. *H*. *pylori-*infected mice had significantly higher scores for inflammation, pseudopyloric metaplasia, and hyperplasia than uninfected mice. Gastric expression of inflammatory cytokines, including TNFα, IL1β, IL7α, and IFNγ, was also significantly upregulated in *H*. *pylori* infected mice compared to uninfected mice, which corroborated histopathologic findings; however, no significant differences were found in inflammatory cytokine expression between *H*. *pylori*-infected mice on ID vs. IR diet. In a related study, *H*. *pylori*-infected gerbils on an ID diet had higher gastric inflammation and dysplasia scores when compared to infected gerbils on an IR diet, and *H*. *pylori* strains isolated from infected and iron-depleted gerbils also demonstrated increased virulence and induction of inflammatory factors *in vitro* when compared to strains isolated from *H*. *pylori* infected gerbils on an IR diet [54]. A different gerbil study found that *H*. *pylori*-infected gerbils had higher gastric pH, higher incidence of gastric ulceration, and higher incidence of fecal occult blood loss, in addition to lower Hgb and MCV than uninfected animals [55].

Hepatic expression of *Hamp*, which encodes the body’s master iron regulatory protein, hepcidin, was significantly lower in *H*. *pylori*-infected mice on ID diet compared to all other treatment groups. An increase in hepcidin production, often related to inflammation, is a defense against bacterial infection[56,57]. In contrast, *Hamp* expression is decreased as a compensatory response in instances when there is a need for increased iron uptake, as seen in cases of IDA [58]. Interestingly, a significant upregulation of hepatic expression of an important iron transport receptor, *Dmt1*, was noted only in the *+Hp*, ID group. High levels of *Dmt1* expression indicate an increased transport of iron into the intracellular space for use in homeostatic functions, and the significant upregulation of expression in the *+Hp*, ID group suggested a more profound systemic need for iron uptake in that group compared to all other treatment groups. The findings related to gene expression in this study highlight a molecular response to low systemic iron status, as determined by low hematocrit, hemoglobin, and ferritin, and provide a basis for further inquiry into the molecular mechanisms of this important response.

We have reconfirmed the findings of previous mouse studies that have evaluated the relationship between *H*. *pylori* infection, ID, and red blood cell homeostasis in mice. *H*. *pylori* infection, coupled with dietary ID, caused anemia and lowered serum iron storage through previously established mechanisms, including loss of gastric parietal cells, change in gastric pH, and inflammation causing upregulation of hepcidin, all resulting in decreased iron absorption and transport. After confirming these findings, we further studied the effects of *H*. *pylori* infection and ID comorbidity on behavior and neuro-homeostasis. We have previously shown that *H*. *pylori* infection in INS-GAS mice altered expression of genes related to iron homeostasis, dopamine metabolism, myelination, and synaptic plasticity in the brain [25]. In the current study, we focused our inquiry on the effects of *H*. *pylori* infection on hippocampal gene expression, and found that infection status had both acute effects, seen in the altered behavior of infected mice in the open field task at 8 weeks postinfection, and chronic effects, in the altered hippocampal gene expression levels in infected mice at 12 months postinfection. The current study found that hippocampal expression of genes related to myelination is lower in in mice chronically infected with *H*. *pylori*, which demonstrates that early-life infection can result in significant alterations to processes that are critical to the maintenance of neurohomeostasis in the adult brain. This study represents the first documentation of *H*. *pylori*-associated inflammation affecting mouse behavioral performances in the open field. Future studies should use gestational and neonatal ID mouse models to more effectively deplete iron during periods of rapid neurodevelopment, which will assist in elucidating the effects of ID and *H*. *pylori* infection comorbidity on the developing brain.
